# Practices and challenges for hemophilia management under resource constraints in Thailand

**DOI:** 10.1186/s13023-023-02718-1

**Published:** 2023-05-10

**Authors:** Chatphatai Moonla, Darintr Sosothikul, Bunchoo Pongtanakul, Bundarika Suwanawiboon, Chanchai Traivaree, Rungrote Natesirinilkul, Nongnuch Sirachainan, Pantep Angchaisuksiri

**Affiliations:** 1 Department of Medicine, Faculty of Medicine, Chulalongkorn University and King Chulalongkorn Memorial Hospital, Thai Red Cross Society, Bangkok, Thailand; 2 Center of Excellence in Translational Hematology, Faculty of Medicine, Chulalongkorn University and King Chulalongkorn Memorial Hospital, Thai Red Cross Society, Bangkok, Thailand; 3 Department of Pediatrics, Faculty of Medicine, Chulalongkorn University and King Chulalongkorn Memorial Hospital, Rama IV Road, Pathumwan, Bangkok, 10330 Thailand; 4 Integrative and Innovative Hematology/Oncology Research Unit, Faculty of Medicine, Chulalongkorn University and King Chulalongkorn Memorial Hospital, Thai Red Cross Society, Bangkok, Thailand; 5 Department of Pediatrics, Faculty of Medicine Siriraj Hospital, Mahidol University, Bangkok, Thailand; 6 Department of Medicine, Faculty of Medicine Siriraj Hospital, Mahidol University, Bangkok, Thailand; 7 Department of Pediatrics, Phramongkutklao College of Medicine and Phramongkutklao Hospital, Bangkok, Thailand; 8 Department of Pediatrics, Faculty of Medicine, Chiang Mai University and Maharaj Nakorn Chiang Mai Hospital, Chiang Mai, Thailand; 9 Department of Pediatrics, Faculty of Medicine Ramathibodi Hospital, Mahidol University, Bangkok, Thailand; 10 Department of Medicine, Faculty of Medicine Ramathibodi Hospital, Mahidol University, Bangkok, Thailand

**Keywords:** Hemophilia, Thailand, Coagulation factor, Clotting factor concentrates, Hemophilia A, Hemophilia B, Healthcare rationing

## Abstract

Hemophilia is an inherited bleeding disorder caused by deficiency of a specific coagulation factor. Factor VIII deficiency is responsible for hemophilia A while factor IX deficiency is responsible for hemophilia B. As per the 2020 annual global survey by the World Federation of Hemophilia, only 1828 Thai hemophiliacs have been registered to the national healthcare system. The reason for the low number is the underdiagnosis which is a major concern in the real-world practice among Asian countries. In Thailand, most hemophiliacs are diagnosed by general practitioners, pediatricians or internists at rural hospitals and are referred to hemophilia specialists at the Hemophilia Treatment Centers (HTCs). Despite the challenges pertaining to infrastructure and cost of treatment, Thailand has progressed substantially in providing the required hemophilia care, as evidenced by an evolution in acquiring and sharing knowledge as well as collaborative efforts among multiple stakeholders over the past three decades. In this letter-to-the-editor, the authors have summarized the practices for and challenges faced with hemophilia management in Thailand.

Hemophilia is an inherited bleeding disorder caused by deficiency of a specific coagulation factor, i.e., factor VIII (FVIII) for hemophilia A (HA) and factor IX (FIX) for hemophilia B (HB) [1]. Due to insufficient intrinsic tenase activity, hemophiliacs tend to bleed into skeletal muscles and major joints, either spontaneously or following trauma/surgery, based upon the disease severity [1]. In the United States, the age-adjusted prevalence of hemophilia is reported to be 15.7 cases/100,000 male individuals. The incidence of HB is approximately 4–6 times lower than that of HA [2]. A substantially lower epidemiology has been documented among 66.1 million Thai population. As per the 2020 annual global survey by the World Federation of Hemophilia (WFH), only 1828 Thai hemophiliacs (1615 of HA and 213 of HB) have been registered to the Thai national healthcare system. Most of them have severe (51.2%) and moderate hemophilia (30.2%) [3]. The reason for the low number is the underdiagnosis which is a major concern in the real-world practice among Asian countries [4]. Mild hemophiliacs are more likely to be unrecognized due to lack of awareness, both medical and self, about non-severe bleeding phenotypes [5].

In Thailand, most hemophiliacs are diagnosed by general practitioners, pediatricians or internists at rural hospitals and are referred to hemophilia specialists at the Hemophilia Treatment Centers (HTCs). Fewer patients are directly diagnosed at the HTCs. Since several hemostatic disorders can share the same clinical spectrum, establishing an accurate diagnosis is crucial [1]. An isolated prolongation of activated partial thromboplastin time (aPTT) accompanied with significant bleeding symptoms may lead to the diagnosis of hemophilia based upon the evidence of FVIII or FIX deficiency from the local laboratories. At the HTCs, referred new cases are retested for FVIII and FIX coagulant activities as well as von Willebrand factor (VWF) levels by the standardized special coagulation laboratories to validate the assigned diagnosis and disease severity. The family members of each proband are usually advised to undergo screening for the factor levels even if they are asymptomatic. Once the diagnosis is confirmed, a prophylactic factor replacement therapy using clotting factor concentrates (CFCs) is generally offered, particularly for severe and moderate hemophiliacs with frequent bleeds. After initiating factor replacement therapy, factor inhibitors should be monitored every 6–12 months due to the risk of inhibitor development in the first 50 exposure days and thereafter [1]. In patients who need surgery, inhibitor assays should be performed preoperatively. Among those with severe HA and/or high-risk *F8* mutations for developing FVIII inhibitors, more frequent inhibitor monitoring is recommended. As reported in 2020, only 5.6% (102/1828) of Thai hemophiliacs had active inhibitors, accounting for 6.2% (100/1615) of HA and 0.9% (2/213) of HB patients [3]. The low inhibitor prevalence among Thai HA population may partially be attributed to a limited accessibility to inhibitor assays in several HTCs.

Prophylactic factor replacement therapy has been recommended as the standard of care for hemophiliacs to minimize risks of bleeding and hemophilic arthropathy [1]. Nevertheless, implementation of adequate prophylaxis remains a major challenge in Thailand due to a limited budget allowance for CFCs (Tables 1, 2). For those requiring prophylactic CFCs more than low-dose practice (10–15 IU/kg for 2–3 times/week) [1], CFC cost exceeds the reimbursable budget and could cause financial burden. Additional challenges faced by the patients and their caregivers include: limited distribution of CFCs and inhibitor testing, increased bleeding risk among adults with hemophilic arthropathy and issues regarding self-infusion of CFCs. Although every hemophiliac and/or caregiver should be trained for venous access and self-infusion, several cases prefer to receive CFC infusions from medical personnel, which can become a hassle for those residing far from the HTCs. The COVID-19 pandemic adversely affected the onsite visits at the HTCs. Therefore, some HTCs alternatively applied telecommunications to help with the follow-ups, referred the patients to the nearest HTC, or delivered CFCs via cold storage to the patients’ residents if feasible. Due to these obstacles, only 38.5% of Thai severe hemophiliacs who regularly used CFC prophylaxis were registered in the 2020 annual global survey (365/947); 67.7% of which were children and teenagers (< 18 years) [3].

**Table 1:** Annual budgets, provided by the Thai National Health Security Office between 2019 and 2022, for prophylactic or on-demand home treatment using clotting factor concentrates in Thai children and adults with hemophilia

Disease severity	Hemophilia A		Hemophilia B	
	Annual budgets per patient	Estimated reimbursable SHL FVIII concentrate units per patient (IU per year)	Annual budgets per patient	Estimated reimbursable SHL FIX concentrate units per patient (IU per year)
Budgets provided by the Thai National Health Security Office				
Children below 10 years of age				
Severe hemophilia	288,000 THB (8180 USD)	28,500–46,000	226,800 THB (6240 USD)	17,500–18,500
Moderate hemophilia	144,000 THB (4090 USD)	14,500–23,000	151,200 THB (4300 USD)	11,500–12,000
Mild hemophilia	36,000 THB (1025 USD)	3750–5750	75,600 THB (2150 USD)	5500–6000
Children above 10 years of age and adults				
Severe hemophilia	345,600 THB (9830 USD)	34,500–55,500	302,400 THB (8590 USD)	23,000–24,000
Moderate hemophilia	144,000 THB (4090 USD)	14,500–23,000	151,200 THB (4300 USD)	11,500–12,000
Mild hemophilia	72,000 THB (2050 USD)	7500–11,500	75,600 THB (2150 USD)	5500–6000

*FVIII* factor VIII, *FIX* factor IX, *IU* international unit, *SHL* standard half-life, *THB* Thai baht, *USD* US dollar (1 USD ≈ 35 THB)

**Table 2:** Annual budgets, provided by the Thai Social Security Office between 2019 and 2022, for prophylactic or on-demand home treatment using clotting factor concentrates in Thai adults with hemophilia

Disease severity	Hemophilia A		Hemophilia B	
	Annual budgets per patient	Estimated reimbursable SHL FVIII concentrate units per patient (IU per year)	Annual budgets per patient	Estimated reimbursable SHL FIX concentrate units per patient (IU per year)
Budgets provided by the Thai Social Security Office^a^				
Severe hemophilia	36,000 IU^b^		30,000 IU^b^	
Moderate hemophilia	18,000 IU^b^		18,000 IU^b^	
Mild hemophilia	6000 IU^b^		6000 IU^b^	

*FVIII* factor VIII, *FIX* factor IX, *IU* international unit, *SHL* standard half-life, *THB* Thai baht, *USD* US dollar (1 USD ≈ 35 THB)

^a^The Thai Social Security Office (SSO) pays for adult patients who are taxable employees and have entitlements to the Thai Social Security Funds

^b^The SSO limits the budgets by the factor amounts (IU) rather than the factor costs

Although prophylactic regimen should be based upon the disease severity and bleeding phenotypes, individualized CFC prophylactic scheme in Thailand is largely influenced by the Thai national budgets for hemophilia care. Since 2019, the Thai National Health Security Office (NHSO) has paid for CFCs, used for both prophylactic/on-demand home treatment (Table 1) and episodic treatment of breakthrough bleeds requiring urgent HTC visits or hospitalization (Table 3). Some adults with hemophilia get reimbursed for these expenses from the Thai Social Security Office (SSO) instead of NHSO (Tables 2, 4). For home treatment in children below 10 years of age, NHSO has provided an annual budget of 288,000 Thai Baht (THB; 8180 USD) and 226,800 THB (6240 USD) per patient for severe HA and HB, respectively. Based upon market prices of CFCs, each patient could receive standard half-life (SHL) FVIII concentrate of 28,500–46,000 IU/year (approximately 500–750 IU/week) or SHL FIX concentrate of 17,500–18,500 IU/year (approximately 500 IU/10 days) for prophylaxis. For children above 10 years of age and adults, the budget allotment from NHSO for home treatment increases to 345,600 THB (9830 USD) and 302,400 THB (8590 USD) per patient for severe HA and HB, respectively. Therefore, SHL FVIII concentrate of 34,500–55,500 IU (approximately 750–1000 IU/week) and SHL FIX concentrate of 23,000–24,000 IU (approximately 500 IU/week), intended for prophylaxis, are reimbursable annually. However, a fixed-dose SHL FVIII concentrate of 500 IU prophylactically given to children with severe HA once weekly does not meet the lowest regimen recommended by the WFH [1], and is insufficient for adults with body weight > 50 kg or advanced-stage hemophilic arthropathy. For severe HB, FIX prophylaxis is more hindered due to a lower budget allocation and a higher cost of SHL FIX concentrate. As a consequence, pediatric hemophiliacs are likely to be undertreated, and joint damages would not be effectively prevented. Although the efficacy data on prophylactic treatment in Thai HB population remain to be determined, a multicenter study on 50 Thai patients with severe HA has suggested that the low-dose prophylaxis using SHL FVIII concentrate of 500 IU twice weekly for 3 months is effective and resulted in zero bleeds in 48% of all subjects and 38% of subjects with preexisting target joints [6]. Future revisions of the Thai national hemophilia budgets should therefore be considered to promote adequate CFC prophylaxis, at least 10 IU/kg twice weekly, and dose escalation should be allowed if needed.

**Table 3:** Clotting factor concentrate budgets, provided by the Thai National Health Security Office between 2019 and 2022, for episodic treatment of breakthrough bleeds requiring urgent outpatient visits or hospitalization in Thai children and adults with hemophilia

Disease severity	Outpatient treatment		Inpatient treatment	
	Budgets per visit^a^	Estimated reimbursable SHL CFCs^b^ (IU per visit)	Budgets per admission	Estimated reimbursable SHL CFCs^b^ (IU per admission)
Budgets provided by the Thai National Health Security Office				
Children in any age groups and adult				
Any severity of hemophilia A	150,000 THB (4260 USD)	15,000–24,000	300,000 THB (8520 USD)	30,000–48,000
Any severity of hemophilia B	150,000 THB (4260 USD)	11,500–12,000	300,000 THB (8520 USD)	23,000–24,000

*CFC* clotting factor concentrate, *IU* international unit, *SHL* standard half-life, *THB* Thai baht, *USD* US dollar (1 USD ≈ 35 THB)

^a^Maximum 2 visits per month

^b^Factor VIII and IX concentrates for patients with hemophilia A and B, respectively

**Table 4:** Clotting factor concentrate budgets, provided by the Thai Social Security Office between 2019 and 2022, for episodic treatment of breakthrough bleeds requiring urgent outpatient visits or hospitalization in Thai adults with hemophilia

Disease severity	Outpatient treatment		Inpatient treatment	
	Budgets per visit^a^	Estimated reimbursable SHL CFCs^b^ (IU per visit)	Budgets per admission	Estimated reimbursable SHL CFCs^b^ (IU per admission)
Budgets provided by the Thai Social Security Office^c^				
Any severity of hemophilia A	15,000 IU^d^		30,000 IU^d^	
Any severity of hemophilia B	15,000 IU^d^		30,000 IU^d^	

*CFC* clotting factor concentrate, *IU* international unit, *SHL* standard half-life, *THB* Thai baht, *USD* US dollar (1 USD ≈ 35 THB)

^a^Maximum 2 visits per month

^b^Factor VIII and IX concentrates for patients with hemophilia A and B, respectively

^c^The Thai Social Security Office (SSO) pays for adult patients who are taxable employees and have entitlements to the Thai Social Security Funds

^d^The SSO limits the budgets by the factor amounts (IU) rather than the factor costs

Recombinant (43.3%) and plasma-derived (50.4%) SHL CFCs have been widely used in Thailand while the availability of extended half-life (EHL) CFCs is limited [3]. As of 2023, recombinant CFCs remain more expensive than plasma-derived CFCs in Thailand (Table 5). Nonacog alpha, a recombinant SHL FIX concentrate which was recently approved by the Thai Food and Drug Administration for HB treatment, has a potential for the low-dose HB prophylaxis (e.g., 500 FIX IU/week) based upon the reimbursable budgets [7], despite the limited local experience. For HA patients with inhibitors, although emicizumab prophylaxis—outside NHSO and SSO coverage—could be used in Thailand, only 7% of patients were able to afford its out-of-pocket costs [3, 8]. Nevertheless, compared with on-demand consumption of bypassing agents during breakthrough bleeding episodes, it is believed that the efficacy of low-dose emicizumab prophylaxis (1 mg/kg emicizumab administered monthly) may reduce the total treatment expenses [8, 9].

**Table 5:** Average market prices of clotting factor concentrate currently available in Thailand

Type of CFC	No. of products available	Dose/vial	Average price per IU^a^ (THB; ± SD)	Average price per IU^a^ (USD; ± SD)
Recombinant SHL FVIII concentrate	3	250 IU 500 IU	9.459 ± 1.744	0.270 ± 0.050
Plasma-derived SHL FVIII concentrate	4	250 IU 500 IU	9.030 ± 1.689	0.258 ± 0.048
Recombinant EHL FVIII concentrate	2	500 IU	9.785 ± 0.049	0.280 ± 0.001
Recombinant SHL FIX concentrate	1	250 IU 500 IU	10.216 ± 0.543	0.292 ± 0.016
Plasma-derived SHL FIX concentrate	2	500 IU 600 IU	10.656 ± 0.410	0.304 ± 0.012

*CFC* clotting factor concentrate, *EHL* extended half-life, *FVIII* factor VIII, *FIX* factor IX, *IU* international unit, *SHL* standard half-life, *THB* Thai baht, *USD* US dollar (1 USD ≈ 35 THB)

^a^Updated data as of April 2023

For social mobilization, the National Hemophilia Foundation of Thailand in cooperation with the Thai Hemophilia Patient Club are actively promoting self-engagement and use of digital technology, i.e., smartwatch monitoring and hemophilia mobile applications, to foster improvement of aerobic exercise capacity and muscle strength among Thai hemophiliacs during the age of digital technology [10]. A study to evaluate the impact of increased physical activities monitored by these modalities during low-dose CFC prophylaxis in Thai hemophiliacs is ongoing (ClinicalTrials.gov NCT05728528). Other innovative technologies, including factor replacement therapy (e.g., EHL CFCs, efanesoctocog alfa), rebalancing non-factor replacement therapy and gene therapy, have a potential role to further improve hemophilia care [11, 12]. Rondaptivon pegol, a VWF-binding aptamer, might be a particularly useful option in supplementing low-dose CFC prophylaxis [13]. Moreover, since the Thai Society of Hematology (TSH) started the national registry of thrombotic and hereditary bleeding disorders in 2016, medical professionals across Thailand could register their patients to collate the real-world hemophilia data. Recently, a sub-registry of hereditary bleeding disorders has been electronically linked with the World Bleeding Disorders Registry endorsed by the WFH. These international collaborative data would hopefully be utilized for the evidence-based advocacy initiatives in the future [14].

## Conclusion

The main limitations of CFC prophylaxis in Thai hemophiliacs are the insufficient budgets, the high costs of CFCs, as well as the lack of infrastructures that help facilitate the treatment. Although the Thai national hemophilia guideline which is currently developed by the TSH would helpfully standardize hemophilia practice in Thailand, more studies are necessary to accurately define the disease burden and the unmet needs for further improvement. Educational initiatives can be planned to promote community awareness and hemophilia screening among at-risk individuals with a family history of hemophilia and/or bleeding symptoms. Moreover, the government should consider increasing the Thai national hemophilia budgets to effectively support prophylactic factor replacement therapy. Despite the challenges, Thailand has progressed substantially in providing the required hemophilia care, as evidenced by an evolution in acquiring and sharing knowledge as well as collaborative efforts between multiple stakeholders over the past 3 decades [6, 8, 9]. Continuous efforts are being made to sustainably improve outcomes of hemophilia treatment, reduce hemophilia-associated morbidity and mortality, and ameliorate quality of life among Thai hemophilia population.

## Data Availability

Not applicable.
